# Lorazepam in Managing Atypical Neuroleptic Malignant Syndrome: A Systematic Review of Case Reports

**DOI:** 10.5811/westjem.41514

**Published:** 2025-08-29

**Authors:** Aaron Chen, Myungwook Bae

**Affiliations:** Maimonides Medical Center, Department of Emergency Medicine, Brooklyn, New York

## Abstract

**Introduction:**

Neuroleptic malignant syndrome (NMS), comprising typical and atypical presentations, is a rare but life-threatening reaction to antipsychotic medications. While typical NMS is characterized by fever, rigidity, and autonomic instability, atypical NMS often lacks these hallmark features, complicating timely diagnosis in the emergency department (ED).

**Methods:**

We conducted a systematic review of PubMed and citation databases (1988–2024) using the keywords “Neuroleptic Malignant Syndrome” and “Lorazepam.” Case reports were screened using strict inclusion criteria and appraised with the Joanna Briggs Institute Checklist.

**Results:**

Lorazepam led to clinical improvement in 5 of 6 atypical and 7 of 9 typical NMS cases. Among five atypical cases, four demonstrated improvements in altered mental status (AMS), resolution of agitation, and reduction in neuromuscular symptoms within 72 hours, including two within 24 hours, compared to the usual 5–10 day recovery with supportive care alone. One atypical case presenting to the ED showed rapid improvement in AMS following early lorazepam administration, although catatonia ultimately necessitated electroconvulsive therapy. These findings highlight lorazepam’s potential benefit in both timely symptom control and diagnostic clarity in atypical NMS.

**Conclusion:**

Lorazepam shows rapid efficacy in atypical NMS, with most cases improving within 72 hours. Yet its subtle presentation often delays diagnosis in the ED, reducing early treatment opportunities. Typical NMS cases demonstrated slower response. Emergency physicians should maintain a high index of suspicion for atypical NMS and consider empiric lorazepam therapy alongside antipsychotic discontinuation and supportive care. Prospective studies are needed to refine ED management strategies.

## INTRODUCTION

Neuroleptic malignant syndrome (NMS) is a rare and potentially fatal idiosyncratic reaction to antipsychotic medications, primarily dopamine antagonists, and disproportionately affect individuals with psychiatric illness. It exists in two recognized forms: typical and atypical NMS. Typical NMS, defined by the *Diagnostic and Statistical Manual of Mental Disorders, 5^th^ Ed* as a drug-induced movement disorder, occurs in 0.01–3.2% of neuroleptic-treated patients.^1^ In contrast, atypical presentation of NMS, known as atypical NMS, offers unique challenges to emergency physicians due to the absence of one or more hallmark features, such as hyperthermia or elevated creatine kinase (CK) levels, making early diagnosis difficult^2^ (Table 1).

### Pathophysiology

First-generation (typical) antipsychotics exert their effects primarily by blocking dopamine receptors in the brain. In contrast, second-generation (atypical) antipsychotics both inhibit dopamine receptors and antagonize serotonin receptors, while modulating norepinephrine and histamine neurotransmission.^3^ The pathophysiology of NMS is largely attributed to the blockade of dopamine D2 receptors in key regions such as the hypothalamus, striatum, and spinal cord, resulting in dysregulation of muscle control, thermoregulation, and autonomic function.^4^

Neuroleptic malignant syndrome has been associated with nearly all antipsychotics. However, high-potency typical antipsychotics are generally considered to pose a greater risk for NMS compared to low-potency atypical antipsychotics. Pharmacodynamic differences between these two classes are notable. Atypical antipsychotics tend to have a lower affinity for dopamine D2 receptors but are potent antagonists of the 5-HT2A serotonin receptor. This higher ratio of 5-HT2A to D2 receptor occupancy is believed to account for the reduced incidence of extrapyramidal side effects seen with atypical antipsychotics.^5^

Evidence from case reports and retrospective studies suggests the existence of atypical NMS, particularly in association with atypical antipsychotics such as quetiapine and clozapine.^6^, ^7^ However, it remains uncertain whether these atypical presentations represent early or impending NMS. Additionally, the potential involvement of other neurotransmitter systems, beyond dopamine, in the pathogenesis of NMS induced by atypical antipsychotics is unclear. Clinicians should be vigilant in assessing for NMS in patients receiving any antipsychotics and should not prematurely rule out the diagnosis when classic features such as severe rigidity or hyperthermia are absent.^8^

### Management and Therapeutic Strategies

Early recognition of atypical NMS in the emergency department (ED) is challenging, as it often requires considerable time to rule out other potential causes of symptoms. Even after excluding other possibilities, emergency physicians may struggle with the diagnosis because atypical NMS often deviates from standard diagnostic criteria and can closely resemble other similar conditions. Overlooking the possibility of atypical NMS from normal lab results could cause delay in withdrawing the offending medication and rapidly lead to severe complications or even death.^9^

Atypical NMS is most commonly associated with atypical antipsychotics, with only a few reported cases of haloperidol-induced atypical NMS in the literature. This increases the likelihood of misdiagnosis in patients taking typical antipsychotics, such as haloperidol.^6^, ^10^, ^11^ Given the medical history and clinical presentation of patients with suspected atypical NMS, the differential diagnosis should include atypical NMS, serotonin syndrome, malignant catatonia, and acute dystonia (Table 2). Seratonin syndrome is a life-threatening, adverse drug reaction caused by excessive serotonergic agonists, with the central triad of symptoms being altered mental status (AMS), autonomic hyperactivity, and neuromuscular abnormalities.^12^ Malignant catatonia is a subtype of catatonia characterized by stupor, mutism, catalepsy, waxy flexibility, negativism, posturing, pyrexia, autonomic dysfunction, rigidity, and elevated creatin kinase (CK) levels.^13^ Acute dystonia, while an emergency, presents as less life-threatening, extrapyramidal adverse effects induced by typical antipsychotics.^14^ Differentiating typical NMS from the other three conditions presents diagnostic challenges. For example, Desai and colleagues reported a case that was initially diagnosed as NMS but later confirmed as malignant catatonia, leading to a change in treatment.^15^ Additionally, patients can develop NMS alongside another condition, complicating diagnosis. Prakash found that NMS and serotonin syndrome can coexist in a single patient, especially when treated with a combination of neuroleptic and serotonergic agents.^16^ Nonetheless, the initial treatment for all three conditions is the same: immediate discontinuation of the causative agents and supportive care.^17^, ^18^

Another challenge associated with treating atypical NMS lies in the realm of pharmacotherapy (Table 3). Benzodiazepines, particularly lorazepam and diazepam, have emerged as an effective treatment option in both typical and atypical NMS. Lorazepam is preferred for its longer intracerebral half-life, higher potency (2–2.5 milligrams [mg] lorazepam equivalent to 10 mg diazepam) and lesser frequency of venous thrombosis.^19^ It exerts its effects by enhancing GABAergic (gamma-aminobutyric acid) transmission, which reduces muscle rigidity and prevents further neuromuscular complications. Intramuscular or intravenous lorazepam are first-line treatments for muscle rigidity and sympathetic overactivity, including agitation and hyperthermia.^20^ Prior literature has demonstrated that all patients who received lorazepam within 24 hours of typical NMS onset or hospital admission experienced resolution of rigidity and fever within 24–48 hours, while other symptoms subsided within 64 hours. This contrasts with recovery periods of 5–10 days in patients who received supportive care alone.^21^ Recent literature supports the use of lorazepam as an effective treatment for atypical NMS as well.^22^, ^23^

In this article we aimed to evaluate the importance of recognizing atypical NMS in emergency settings and lorazepam’s role as a frontline treatment by analyzing published reports of NMS. Identifying atypical cases within the broader pool of NMS reports helps explore the nuances of recognizing atypical NMS and compare clinical responses to lorazepam between typical and atypical presentations. This approach both highlights the diagnostic challenges of atypical NMS in emergency settings and contributes to understanding lorazepam’s efficacy across the clinical spectrum of NMS.

## METHODS

We performed a systematic literature search in adherence to the Preferred Reporting Items for Systematic Reviews and Meta-Analyses (PRISMA) protocol. PubMed was queried for studies published between 1988–December 6, 2024, using the key terms “Neuroleptic Malignant Syndrome” and “Lorazepam.” To enhance the yield of relevant studies, we also performed a citation-based extension search by reviewing reference lists and citations of included articles.

### Eligibility Criteria

Given the rarity of NMS and the predominance of case reports in the available literature, studies were included if they met the following inclusion criteria:

1. Case reports or case series with a final diagnosis of NMS
2. Use of lorazepam as a primary treatment for NMS
3. Clear documentation of clinical outcomes following lorazepam administration
4. Papers published in English with accessible abstracts and full texts.

Studies were excluded based on the following exclusion criteria:

1. Papers not written in English
2. Papers lacking access to abstracts or full texts
3. NMS not being the final diagnosis
4. Treatment involved other medications either in place of or in combined with lorazepam.

We identified atypical NMS cases among eligible reports and analyzed them separately. The primary outcomes assessed included the number of patients and their clinical responses to lorazepam treatment.

### Risk-of-bias Assessment

We used the Joanna Briggs Institute (JBI) Critical Appraisal Checklist for Case Reports to assess the risk of bias in the included case reports and case series (https://jbi.global/critical-appraisal-tools). Only studies that fulfilled all eight criteria on the checklist were deemed eligible for final analysis.

## RESULTS

### Study Selection

**Figure 1:**
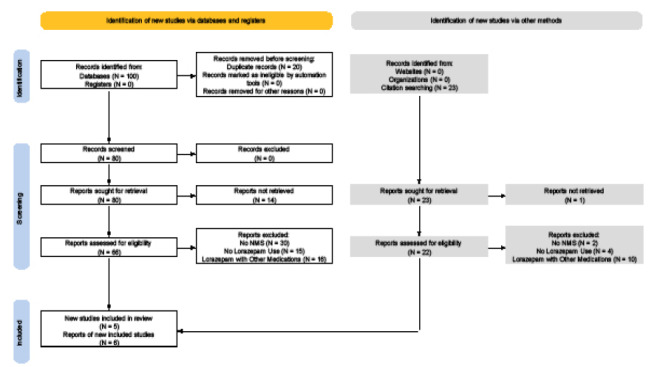
PRISMA flow chart outlining inclusion and exclusion criteria for articles that referenced use of lorazepam. *PRISMA*, Preferred Reporting Items for Systematic Reviews and Meta-Analyses; *NMS*, neuroleptic malignant syndrome.

The PubMed search identified 100 articles, with an additional 23 identified from citation searches. We inititally excluded 20 papers for the following reasons: five due to duplication; and 15 papers (14 in PubMed and one in citation search) not written in English or lacking an abstract or access to full text. An additional 77 studies were excluded (61 in PubMed and 16 in citation search for NMS-irrelevant diagnoses) because lorazepam had not been administered or more than lorazepam had been administered (Figure 1). Screening with the JBI checklist resulted in a total of 11 articles, five from the PubMed and six from citation search, comprising a total of 15 cases.

### Patient Characteristics and Treatment Outcomes

Among the 11 articles reviewed, we identified a total of 15 cases (10 males and 5 females 17–83 years of age). Our cohort consisted of six atypical and nine typical NMS cases, in which lorazepam administration showed a significant improvement in 12 of 15 cases (Table 4). Among atypical NMS cases, 5 of 6 patients demonstrated a favorable response to lorazepam: two improved significantly within 24 hours; two within 72 hours; and one case that lacked a specified recovery timeline. In the remaining case, lorazepam appeared to initially improve mental status but failed to resolve catatonia, which ultimately required electroconvulsive therapy (ECT). On the other hand, among typical NMS cases, 7 of 9 patients showed clinical improvement following lorazepam therapy. Only two patients were reported to have presented to the ED, and they both exhibited progressive improvement post-administration, consistent with the other five inpatient cases. Of the seven who responded favorably to treatment with lorazepam, three demonstrated significant improvement within 72 hours, two recovered over an extended course, and two lacked documented timelines.

## DISCUSSION

The rarity of typical NMS, combined with the lack of clinical trials, makes treatment challenging, as most drug recommendations are based on pharmacokinetic theories and clinical observations.^35^ Given that atypical NMS is even rarer, its treatment is likely to be even more difficult. A step-by-step treatment approach has been proposed based on the severity of NMS, addressing both typical and atypical cases (Table 5). The Woodbury stage approach suggests that lorazepam may serve as a first-line pharmacologic intervention in stages II, III or IV, where patients present with mild or moderate symptoms. The Woodbury approach also suggests other commonly used medications, such as bromocriptine, dantrolene, and amantadine in advanced stages.^36^ However, it is important to note that the effectiveness of these drugs varies in different cases, and none have yet received approval from the US Food and Drug Administration. Our literature review reinforces these findings, with 12 of 15 patients with NMS demonstrating significant clinical improvement following lorazepam administration. When comparing outcomes between atypical and typical NMS cases, 5 of 6 atypical cases (83%) and 7 of 9 typical cases (78%) responded favorably to lorazepam. Notably, 4 of 6 atypical cases (67%) and 3 of 9 typical cases (33%) exhibited significant clinical improvement within 72 hours of lorazepam initiation. These results suggest that lorazepam may expedite hospital course by facilitating meaningful symptom resolution within 72 hours in most atypical NMS cases and approximately one-third of typical cases.

All six atypical NMS cases initially presented to the ED with AMS in the absence of fever. Only one case received timely intervention in the ED, which included discontinuation of antipsychotics, supportive care, and administration of lorazepam. It was also the only instance in which lorazepam alone proved insufficient. Although the patient’s AMS improved following initial lorazepam administration in the ED, persistent catatonia ultimately necessitated treatment with ECT during admission. Of note, this patient had previously responded favorably to parenteral lorazepam during an earlier episode of prodromal NMS in the ED, underscoring lorazepam’s potential role in addressing early neuroleptic toxicity—even if incomplete in complex or refractory presentations.

In contrast, the other five atypical NMS patients, although diagnosed later during hospitalization, demonstrated substantial clinical improvement following lorazepam initiation, typically within 72 hours. This delay highlights the diagnostic challenges of atypical NMS in emergency settings, where its subtle and non-classic presentation may hinder early recognition, and suggests that prompt administration of lorazepam may offer a valuable therapeutic strategy for reversing key symptoms and improving patient outcomes.

## LIMITATIONS

Given the rarity of NMS, our review is primarily based on case reports and case series, which inherently have certain limitations. These include a lack of generalizability, potential overestimation of lorazepam’s efficacy, selection bias, limited statistical power, and the influence of confounding variables such as patients’ comorbidities. Additionally, most reports lacked precise timelines, limiting our ability to assess the onset of lorazepam’s effect at the ED level. While some cases suggested early improvement, this could not be quantified. Future clinical trials are needed to validate lorazepam’s efficacy in both atypical and typical NMS.

## CONCLUSION

Early recognition and prompt intervention for atypical neuroleptic malignant syndrome remain critical challenges in emergency medicine due to its subtle and varied clinical presentations. Our systematic review underscores lorazepam’s effectiveness as a first-line pharmacologic intervention, demonstrating significant symptomatic improvement within 72 hours in most atypical NMS cases (4 of 6), including two within 24 hours. This result is comparable or superior to outcomes observed in typical presentations (3 of 9). Notably, one of the two atypical NMS cases that did not fully respond to lorazepam received early administration of lorazepam in the ED and substantially improved the degree of AMS prior to admission, which reinforced the potential benefits of timely therapy. Future controlled studies are essential to refine diagnostic criteria and treatment protocols for this potentially lethal condition.

## Figures and Tables

**Table 1:** Comparison of typical and atypical neuroleptic malignant syndrome.

	Typical NMS	Atypical NMS
Exclusions	The symptoms are not due to other substances or medical conditions.	
Risk factor	Expose to dopamine antagonist within 72 hours prior to the beginning of symptoms.	
Major symptoms	Hyperthermia (> 38°C, measured 2 times) Muscle rigidity (generalized, severe)	Often absent or mild. Muscle rigidity (mild to moderate)
Minor symptoms	Autonomic nerve system: Tachycardia Hypertonia Sialorrhea Urinary incontinence Tachypnea Dyspnea Diaphoresis Mental Status: AMS Stupor to coma Motor symptoms: Tremor Akinesia Dystoria Myoclonia Trismus Dysarthria Dysphagia Lab Finding: CK↑ Leukocytes↑ Myoglobin↑ Catecholamines↑ Creatinine↑ Metabolic acidosis Hypoxia	Autonomic nerve system: Present, but less severe Mental status: Less severe Motor symptoms: Present, less severe Lab finding: May present mildly abnormal, but often normal

↑, elevated levels.

*NMS*, neuroleptic malignant syndrome; *AMS*, altered mental status; *CK*, creatine kinase.

**Table 2:** Differential diagnoses of atypical neuroleptic malignant syndrome,

Differential diagnosis	Key clinical presentations	Diagnostic criteria	Treatments
Atypical NMS	Muscle rigidity AMS	Clinical presentations History of antipsychotic use	Discontinuation of antipsychotic use Supportive care Lorazepam Dantrolene
Serotonin syndrome	Hyperreflexia Clonus AMS	Clinical presentations History of serotonergic agent use	Discontinuation of antipsychotic use Supportive care Lorazepam Cyproheptadine
Malignant catatonia	Catatonic behavior Waxy flexibility Autonomic instability	Clinical presentations EEG findings	Lorazepam ECT
Acute dystonia	Involuntary muscle contractions. Mental status preserved.	Clinical presentations	Benztropine Diphenhydramine

*NMS*, neuroleptic malignant syndrome; *AMS*, altered mental status; *EEG*, electroencephalogram; *ECT*, electroconvulsive therapy.

**Table 3:** Pharmacologic treatment options for neuroleptic malignant syndrome.

	Mechanism	Recommended dose	Therapeutic effects
Benzodiazepines: Lorazepam Diazepam	Enhanced GABAergic transmission	Lorazepam: 1–2 mg IM or IV every 4–6 hours. Diazepam: 10mg IV every 8 hours.	Treat agitation Muscle relaxation Reduce seizure risk
Bromocriptine	Dopamine agonist	2.5 mg every 6–8 hours and titrated up to a maximum dose of 40 mg/day.	Muscle relaxation Treat hyperthermia Improve mental status
Amantadine	Dopamine and anticholinergic effects	100–200 mg initially and is titrated upward as needed to a maximum dose of 200 mg every 12 hours.	Muscle relaxation Alleviate tremors Improve mental status

*GABA*, gamma-aminobutyric acid; *mg*, milligram; *IM*, intramuscular; *IV*, intravenous.

**Table 4:** PRISMA search resulting in findings of 12/15 patients experiencing improvement with lorazepam administration.

Search results	Patient demographics	Diagnosis	Presenting complaint and diagnostic consideration	Lorazepam use and outcome
Allonce J et al 2024^24^	43-year-old male	Atypical NMS	Presented with AMS and afebrile. Atypical NMS was diagnosed.	Lorazepam improved muscle rigidity and recovery in 48 hours.
Verma K et al 2018^25^	69-year-old male	Atypical NMS	Presented with AMS and afebrile, agitation, catatonia. Atypical NMS was diagnosed.	Lorazepam seems to have improved AMS prior to admission but did not improve catatonia.
Hernandez SD et al 2021^26^	44-year-old male	Atypical NMS	Presented with AMS and afebrile. Alcoholic encephalopathy secondary to alcohol intoxication was made as initial diagnosis.	Lorazepam resolved agitation.
Mayur Pandya, et al. 2004^27^	28-year-old female	Atypical NMS	Presented with AMS and afebrile. Atypical NMS was diagnosed.	Lorazepam improved AMS and tremors within 72 hours.
	67-year-old female	Atypical NMS	Presented with AMS, afebrile and catatonia. Atypical NMS was diagnosed.	Lorazepam improved AMS within 72 hours.
	83-year-old male	Atypical NMS	Presented with AMS and afebrile with normal CK. Atypical NMS was diagnosed.	Lorazepam improved muscle rigidity, gait, and AMS in 48 hours.
Esang M et al 2019^28^	59-year-old male	Typical NMS	Typical NMS presentation in the ED; diagnosis made based on standard symptom constellation.	Lorazepam led to symptom resolution; stable at discharge.
Velosa A et al 2019^29^	44-year-old female	Typical NMS	Typical NMS presentation; diagnosis made based on standard symptom constellation.	Lorazepam produced no significant improvement. ECT needed
Yacoub A, et al 2006^30^	66-year-old male	Typical NMS		Lorazepam improved muscle rigidity and fever within 48 hours. All other NMS manifestations resolved in 9 days
	62-year-old male	Typical NMS	Typical NMS presentation; diagnosis made based on standard symptom constellation.	Lorazepam improved muscle rigidity and fever within 72 hours. All other features resolved in 5 days.
	43-year-old male	Typical NMS		Lorazepam improved muscle rigidity and fever within 72 hours. All other features resolved in 9 days.
Rosebush P et al 2008^31^	17-year-old male	Typical NMS	Typical NMS presentation; diagnosis made based on standard symptom constellation.	Lorazepam resulted in substantial recovery by day 6 and full recovery by day 14.
Ghio L et al 2009^32^	45-year-old female	Typical NMS	Typical NMS presentation; diagnosis made based on standard symptom constellation.	Lorazepam progressively improved muscle rigidity.
Reilly T et al 2017^33^	49-year-old male	Typical NMS	Typical NMS presentation in the ED; diagnosis made based on standard symptom constellation.	Lorazepam gradually improved muscle rigidity and communication.
Foguet-Boreu Q et al 2018^34^	67-year-old female	Typical NMS	Typical NMS presentation; diagnosis made based on standard symptom constellation.	A trial with lorazepam was not successful. AMS continued. ECT and bromocriptine started for catatonia.

*NMS*, neuroleptic malignant syndrome; *AMS, altered mental status; CK*, creatine kinase; *ED*, emergency department; *ECT*, electroconvulsive therapy.

**Table 5:** Woodbury stages for clinical presentation and treatment of neuroleptic malignant syndrome.

Woodbury Stage	Stage I Drug-induced Parkinsonism	Stage II Drug-induced Catatonia	Stage III Mild, early NMS	Stage IV Moderate NMS	Stage V Severe NMS
Clinical presentation	Rigidity Tremor	Rigidity Mutism Stupor	Mild rigidity Catatonia or confusion Temperature ≤ 38°C (100.4°F) Heart rate ≤100 bpm	Moderate rigidity Catatonia or confusion Temperature 38–40°C (100.4–104°F) Heart rate 100–120 bpm	Severe rigidity Catatonia or coma Temperature ≥ 40°C (104°F) Heart rate ≥ 120 bpm
Supportive care	Reduce or switch antipsychotics	Discontinue, reduce, or switch antipsychotic	Discontinue antipsychotics Carefully monitor for progression Correct risk factors	Discontinue antipsychotics Manage fluids Initiate cooling Correct risk factors Provide intensive care	Discontinue antipsychotics Manage fluids Initiate cooling Correct risk factors Provide intensive care
First-line intervention	Anticholinergic agents	Lorazepam	Lorazepam	Lorazepam, bromocriptine, or amantadine	Dantrolene, bromocriptine, or amantadine
Second-line intervention				Consider ECT	Consider ECT

*NMS*, neuroleptic malignant syndrome; *bpm*, beats per minute; *°C*, degrees Celsius; *°F*, Farenheit; *bpm*, beats per minute; *ECT*, electroconvulsive therapy
